# The wondrous world of biology

**DOI:** 10.4102/sajid.v37i2.372

**Published:** 2022-02-14

**Authors:** Penny Moore

**Affiliations:** 1 SAMRC Antibody Immunity Research Unit, Faculty of Health Sciences, University of the Witwatersrand, Johannesburg, South Africa; 2 National Institute for Communicable Diseases, Johannesburg, South Africa

I am the South African Research Chair of Virus Host Dynamics, and I am based at the National Institute for Communicable Diseases (NICD) and the University of the Witwatersrand (WITS). My early training in microbiology was at WITS, where I completed my BSc (Hons) and MSc, after which I went to the University of London to pursue my PhD on hepatitis B virus. In 2003, I returned to South Africa to join the NICD, where I have been based ever since. I have always been interested in biology, having been raised by parents who were very much aware of nature. We spent a lot of time outdoors, chasing butterflies and watching birds. I also benefited enormously from truly wonderful schoolteachers who made me fall in love with biology very early on. However, my interest in virology probably started when, as a family, we became infected with the hepatitis A virus. My mother was only slightly ill, I was very ill for many weeks but my father died. I found it fascinating that one virus could have such divergent outcomes and since then, I have been hooked on viruses.

Our research has been very focused on the design of HIV vaccine, as South Africa bears the brunt of the HIV pandemic, being home to 7.5 million people with HIV infection. I have had the privilege of being part of The Centre for the AIDS Program of Research in South Africa (CAPRISA), led by Salim Abdool Karim, since 2003. This study focuses on a cohort of young women with HIV infection who live in KwaZulu-Natal province, South Africa, where HIV infection remains rampant, especially in young women. We have been studying women in this cohort since they became infected, some 15 years ago, and have stored blood specimens every couple of months over this period. This enables us to look back in time, immunologically, and understand how some people make good antibodies, capable of recognising many HIV strains, and others do not. Understanding this enables us to design vaccine strategies that have good responses, which we believe are essential for an effective HIV vaccine. We have also isolated monoclonal antibodies from some of these women, one of which is the most potent antibody in clinical development for HIV prevention. This antibody is currently being evaluated in women with no HIV infection, and shows a safe profile. Similar studies to assess how well this antibody will protect infants born to HIV-positive mothers will start soon. I am glad to have been part of developing an antibody that will prevent infection of adults and infants at risk of HIV infection – this is the kind of public health impact that makes science wonderful.

Since the emergence of severe acute respiratory syndrome coronavirus 2 (SARS-CoV-2), my team has expanded to work on this virus too. We rapidly adapted our existing platforms to understand SARS-CoV-2 infection and vaccination, and we were amongst the first laboratories to identify the Beta variant as being antibody resistant. We have made many contributions to understanding the immune response to SARS-CoV-2, including in the context of HIV infection, which results in impaired immunity to coronavirus disease 2019 (COVID-19). Our work has unravelled the responses to vaccination, showed that previous infection dramatically boosts these responses and defined what happens to vaccinated people who become infected. We have also demonstrated that not all SARS-CoV-2 variants are equally able to trigger good antibodies, which has implications in designing second-generation vaccine candidates. This work has been done at an unprecedented pace, and I am hugely proud of the commitment and dedication my team showed in taking on this new challenge while still doing HIV research. HIV research benefitted our SARS-CoV-2 research. Now, we need to translate that urgency back into HIV research.

I have benefitted from amazing mentors. Jen Alexander triggered my interest in research. Lynn Morris has been an astonishingly generous, wonderful mentor over nearly two decades, and Carolyn Williamson has shaped my science significantly and has been a constant reminder that family life and science can happen simultaneously. I love doing research. It’s creative and imaginative, and impactful. It also allows me to train and mentor others. As the daughter of a teacher, this is hugely important to me. I have an incredibly bright, committed team whose members surprise me every day with their ideas and passion. So, my advice to colleagues who want to achieve a fulfilling career in the field of infectious diseases is to surround yourself with smart, talented and big-hearted people, and have fun. Science is not the easiest career, and it requires determination, but it’s worth it!

